# The declaration of Istanbul on organ trafficking and transplant tourism

**DOI:** 10.4103/0971-4065.43686

**Published:** 2008-07

**Affiliations:** Participants in the International Summit on Transplant Tourism and Organ Trafficking convened by The Transplantation Society and International Society of Nephrology in Istanbul, Turkey, April 30–May 2, 2008*

## Preamble

Organ transplantation, one of the medical miracles of the twentieth century, has prolonged and improved the lives of hundreds of thousands of patients worldwide. The many great scientific and clinical advances of dedicated health professionals, as well as countless acts of generosity by organ donors and their families, have made transplantation not only a life-saving therapy but a shining symbol of human solidarity. Yet these accomplishments have been tarnished by numerous reports of trafficking in human beings who are used as sources of organs and of patient-tourists from rich countries who travel abroad to purchase organs from poor people. In 2004, the World Health Organization, called on member states “to take measures to protect the poorest and vulnerable groups from transplant tourism and the sale of tissues and organs, including attention to the wider problem of international trafficking in human tissues and organs”.^1^

To address the urgent and growing problems of organ sales, transplant tourism and trafficking in organ donors in the context of the global shortage of organs, a Summit Meeting of more than 150 representatives of scientific and medical bodies from around the world, government officials, social scientists, and ethicists, was held in Istanbul from April 30 to May 2, 2008. Preparatory work for the meeting was undertaken by a Steering Committee convened by The Transplantation Society (TTS) and the International Society of Nephrology (ISN) in Dubai in December 2007. That committee's draft declaration was widely circulated and then revised in light of the comments received. At the Summit, the revised draft was reviewed by working groups and finalized in plenary deliberations.

This Declaration represents the consensus of the Summit participants. All countries need a legal and professional framework to govern organ donation and transplantation activities, as well as a transparent regulatory oversight system that ensures donor and recipient safety and the enforcement of standards and prohibitions on unethical practices.

Unethical practices are, in part, an undesirable consequence of the global shortage of organs for transplantation. Thus, each country should strive both, to ensure that programs to prevent organ failure are implemented and to provide organs to meet the transplant needs of its residents from donors within its own population or through regional cooperation. The therapeutic potential of deceased organ donation should be maximized not only for kidneys but also for other organs, appropriate to the transplantation needs of each country. Efforts to initiate or enhance deceased donor transplantation are essential to minimize the burden on living donors. Educational programs are useful in addressing the barriers, misconceptions and mistrust that currently impede the development of sufficient deceased donor transplantation; successful transplant programs also depend on the existence of the relevant health system infrastructure.

Access to healthcare is a human right but often not a reality. The provision of care for living donors before, during and after surgery–as described in the reports of the international forums organized by TTS in Amsterdam and Vancouver^2^–^4^ is no less essential than taking care of the transplant recipient. A positive outcome for a recipient can never justify harm to a live donor; on the contrary, for a transplant with a live donor to be regarded as a success means that both the recipient and the donor have done well.

This Declaration builds on the principles of the Universal Declaration of Human Rights.^5^ The broad representation at the Istanbul Summit reflects the importance of international collaboration and global consensus to improve donation and transplantation practices. The Declaration will be submitted to relevant professional organizations and to the health authorities of all countries for consideration. The legacy of transplantation must not be the impoverished victims of organ trafficking and transplant tourism but rather a celebration of the gift of health by one individual to another.

## Definitions

**Organ trafficking** is the recruitment, transport, transfer, harboring or receipt of living or deceased persons or their organs by means of the threat or use of force or other forms of coercion, of abduction, of fraud, of deception, of the abuse of power or of a position of vulnerability, or of the giving to, or the receiving by, a third party of payments or benefits to achieve the transfer of control over the potential donor, for the purpose of exploitation by the removal of organs for transplantation.^6^

**Transplant commercialism** is a policy or practice in which an organ is treated as a commodity, including by being bought or sold or used for material gain.

**Travel for transplantation** is the movement of organs, donors, recipients or transplant professionals across jurisdictional borders for transplantation purposes. Travel for transplantation becomes transplant tourism if it involves organ trafficking and/or transplant commercialism or if the resources (organs, professionals and transplant centers) devoted to providing transplants to patients from outside a country undermine the country's ability to provide transplant services for its own population.

## Principles

1. National governments, working in collaboration with international and non-governmental organizations, should develop and implement comprehensive programs for the screening, prevention and treatment of organ failure, which include:
  - The advancement of clinical and basic science research;
  - Effective programs, based on international guidelines, to treat and maintain patients with end-stage diseases, such as dialysis programs for renal patients, to minimize morbidity and mortality, alongside transplant programs for such diseases;
  - Organ transplantation as the preferred treatment for organ failure for medically suitable recipients.
2. Legislation should be developed and implemented by each country or jurisdiction to govern the recovery of organs from deceased and living donors and the practice of transplantation, consistent with international standards.
  - Policies and procedures should be developed and implemented to maximize the number of organs available for transplantation, consistent with these principles;
  - The practice of donation and transplantation requires oversight and accountability by health authorities in each country to ensure transparency and safety;
  - Oversight requires a national or regional registry to record deceased and living donor transplants;
  - Key components of effective programs include public education and awareness, health professional education and training, and defined responsibilities and accountabilities for all stakeholders in the national organ donation and transplant system.
3. Organs for transplantation should be equitably allocated within countries or jurisdictions to suitable recipients without regard to gender, ethnicity, religion, or social or financial status.
  - Financial considerations or material gain of any party must not influence the application of relevant allocation rules.
4. The primary objective of transplant policies and programs should be optimal short- and long-term medical care to promote the health of both donors and recipients.
  - Financial considerations or material gain of any party must not override primary consideration for the health and well-being of donors and recipients.
5. Jurisdictions, countries and regions should strive to achieve self-sufficiency in organ donation by providing a sufficient number of organs for residents in need from within the country or through regional cooperation.
  - Collaboration between countries is not inconsistent with national self- sufficiency as long as the collaboration protects the vulnerable, promotes equality between donor and recipient populations, and does not violate these principles;
  - Treatment of patients from outside the country or jurisdiction is only acceptable if it does not undermine a country's ability to provide transplant services for its own population.
6. Organ trafficking and transplant tourism violate the principles of equity, justice and respect for human dignity and should be prohibited. Because transplant commercialism targets impoverished and otherwise vulnerable donors, it leads inexorably to inequity and injustice and should be prohibited. In Resolution 44.25, the World Health Assembly called on countries to prevent the purchase and sale of human organs for transplantation.
  - Prohibitions on these practices should include a ban on all types of advertising (including electronic and print media), soliciting, or brokering for the purpose of transplant commercialism, organ trafficking, or transplant tourism.
  - Such prohibitions should also include penalties for acts—such as medically screening donors or organs, or transplanting organs—that aid, encourage, or use the products of, organ trafficking or transplant tourism.
  - Practices that induce vulnerable individuals or groups (such as illiterate and impoverished persons, undocumented immigrants, prisoners, and political or economic refugees) to become living donors are incompatible with the aim of combating organ trafficking, transplant tourism and transplant commercialism.

## Proposals

Consistent with these principles, participants in the Istanbul Summit suggest the following strategies to increase the donor pool and to prevent organ trafficking, transplant commercialism and transplant tourism and to encourage legitimate, life-saving transplantation programs:

To respond to the need to increase deceased donation:

1. Governments, in collaboration with health care institutions, professionals, and non-governmental organizations should take appropriate actions to increase deceased organ donation. Measures should be taken to remove obstacles and disincentives to deceased organ donation.
2. In countries without established deceased organ donation or transplantation, national legislation should be enacted that would initiate deceased organ donation and create transplantation infrastructure, so as to fulfill each country's deceased donor potential.
3. In all countries where deceased organ donation has been initiated, the therapeutic potential of deceased organ donation and transplantation should be maximized.
4. Countries with well established deceased donor transplant programs are encouraged to share information, expertise and technology with countries seeking to improve their organ donation efforts.

To ensure the protection and safety of living donors and appropriate recognition for their heroic act while combating transplant tourism, organ trafficking and transplant commercialism:

1. The act of donation should be regarded as heroic and honored as such by representatives of the government and civil society organizations.
2. The determination of the medical and psychosocial suitability of the living donor should be guided by the recommendations of the Amsterdam and Vancouver Forums.^2^–^4^
  - Mechanisms for informed consent should incorporate provisions for evaluating the donor's understanding, including assessment of the psychological impact of the process;
  - All donors should undergo psychosocial evaluation by mental health professionals during screening.
3. The care of organ donors, including those who have been victims of organ trafficking, transplant commercialism, and transplant tourism, is a critical responsibility of all jurisdictions that sanctioned organ transplants utilizing such practices.
4. Systems and structures should ensure standardization, transparency and accountability of support for donation.
  - Mechanisms for transparency of process and follow-up should be established;
  - Informed consent should be obtained both for donation and for follow-up processes.
5. Provision of care includes medical and psychosocial care at the time of donation and for any short- and long-term consequences related to organ donation.
  - In jurisdictions and countries that lack universal health insurance, the provision of disability, life, and health insurance related to the donation event is a necessary requirement in providing care for the donor;
  - In those jurisdictions that have universal health insurance, governmental services should ensure donors have access to appropriate medical care related to the donation event;
  - Health and/or life insurance coverage and employment opportunities of persons who donate organs should not be compromised;
  - All donors should be offered psychosocial services as a standard component of follow-up;
  - In the event of organ failure in the donor, the donor should receive:
    - Supportive medical care, including dialysis for those with renal failure, and
    - Priority for access to transplantation, integrated into existing allocation rules as they apply to either living or deceased organ transplantation.
6. Comprehensive reimbursement of the actual, documented costs of donating an organ does not constitute a payment for an organ, but is rather part of the legitimate costs of treating the recipient.
  - Such cost-reimbursement would usually be made by the party responsible for the costs of treating the transplant recipient (such as a government health department or a health insurer);
  - Relevant costs and expenses should be calculated and administered using transparent methodology, consistent with national norms;
  - Reimbursement of approved costs should be made directly to the party supplying the service (such as to the hospital that provided the donor's medical care);
  - Reimbursement of the donor's lost income and out-of-pockets expenses should be administered by the agency handling the transplant rather than paid directly from the recipient to the donor.
7. Legitimate expenses that may be reimbursed when documented include:
  - The cost of any medical and psychological evaluations of potential living donors who are excluded from donation (e.g. because of medical or immunologic issues discovered during the evaluation process);
  - Costs incurred in arranging and effecting the pre-, peri- and post-operative phases of the donation process (e.g. long-distance telephone calls, travel, accommodation and subsistence expenses);
  - Medical expenses incurred for post-discharge care of the donor;
  - Lost income in relation to donation (consistent with national norms).

## The participants in the international summit on transplant tourism and organ trafficking and the manner in which they were chosen and the meeting was organized were as follows:

The Steering Committee was selected by an Organizing Committee consisting of Mona Alrukhami, Jeremy Chapman, Francis Delmonico, Mohamed Sayegh, Faissal Shaheen, and Annika Tibell.

The Steering Committee was composed of leadership from The Transplantation Society, including its President-elect and the Chair of its Ethics Committee, and the International Society of Nephrology, including its Vice President and individuals holding Council positions. The Steering Committee had representation from each of the continental regions of the globe with transplantation programs.

The mission of the Steering Committee was to draft a Declaration for consideration by a diverse group of participants at the Istanbul Summit. The Steering Committee also had the responsibility to develop the list of participants to be invited to the Summit meeting.

**Istanbul participant selection:**

Participants at the Istanbul Summit were selected by the Steering Committee according to the following considerations:

- The country liaisons of The Transplantation Society representing virtually all countries with transplantation programs;
- Representatives from international societies and the Vatican;
- Individuals holding leadership positions in nephrology and transplantation;
- Stakeholders in the public policy aspect of organ transplantation; and
- Ethicists, anthropologists, sociologists, and legal scholars wellrecognized for their writings regarding transplantation policy and practice.

No person or group was polled with respect to their opinion, practice, or philosophy prior to the Steering Committee selection or the Istanbul Summit.

After the proposed group of participants was prepared and reviewed by the Steering Committee, they were sent a letter of invitation to the Istanbul Summit, which included the following components:

- the mission of the Steering Committee to draft a Declaration for all Istanbul participants' consideration;
- the agenda and work group format of the Summit;
- the procedure for the selection of participants;
- the work group topics;
- an invitation to the participants to indicate their work group preferences;
- the intent to communicate a draft and other materials before the Summit convened;
- the Summit goals to assemble a final Declaration that could achieve consensus and would address the issues of organ trafficking, transplant tourism and commercialism, and provide principles of practice and recommended alternatives to address the shortage of organs;
- an acknowledgment of the funding provided by Astellas Pharmaceuticals for the Summit;
- provision of hotel accommodations and travel for all invited participants.

Of approximately 170 persons invited, 160 agreed to participate and 152 were able to attend the Summit in Istanbul on April 30-May 2, 2008. Because work on the Declaration at the Summit was to be carried out by dividing the draft document into separate parts, Summit invitees were assigned to a work group topic based on their response concerning the particular topics on which they wished to focus their attention before and during the Summit.

**Preparation of the declaration:**

The draft Declaration prepared by the Steering Committee was furnished to all participants with ample time for appraisal and response prior to the Summit. The comments and suggestions received in advance were reviewed by the Steering Committee and given to leaders of the appropriate work group at the Summit. (Work group leaders were selected and assigned from the Steering Committee.)

The Summit meeting was formatted so that breakout sessions of the work groups could consider the written responses received from participants prior to the Summit as well as comments from each of the work group participants. The work groups elaborated these ideas as proposed additions to and revisions of the draft. When the Summit reconvened in plenary session, the Chairs of each work group presented the outcome of their breakout session to all Summit participants for discussion. During this process of review, the wording of each section of the Declaration was displayed on a screen before the plenary participants and was modified in light of their comments until a consensus was reached on each point.

The content of the Declaration is derived from the consensus that was reached by the participants at the Summit in the plenary sessions which took place on May 1 and 2, 2008. A formatting group was assembled immediately after the Summit to address punctuation, grammatical and related concerns and to record the Declaration in its finished form.

**Table:** Participants in the Istanbul Summit

Last name	First name	Country
Abboud	Omar	Sudan
* Abbud-Filho	Mario	Brazil
Abdramanov	Kaldarbek	Kyrgyzstan
Abdulla	Sadiq	Bahrain
Abraham	Georgi	India
Abueva	Amihan V.	Philippines
Aderibigbe	Ademola	Nigeria
* Al-Mousawi	Mustafa	Kuwait
Alberu	Josefina	Mexico
Allen	Richard D.M.	Australia
Almazan-Gomez	Lynn C.	Philippines
Alnono	Ibrahim	Yemen
* Alobaidli	Ali Abdulkareem	United Arab Emirates
* Alrukhaimi	Mona	United Arab Emirates
Álvarez	Inés	Uruguay
Assad	Lina	Saudi Arabia
Assounga	Alain G.	South Africa
Baez	Yenny	Colombia
* Bagheri	Alireza	Iran
* Bakr	Mohamed Adel	Egypt
Bamgboye	Ebun	Nigeria
* Barbari	Antoine	Lebanon
Belghiti	Jacques	France
Ben Abdallah	Taieb	Tunisia
Ben Ammar	Mohamed Salah	Tunisia
Bos	Michael	The Netherlands
Britz	Russell	South Africa
Budiani	Debra	USA
* Capron	Alexander	USA
Castro	Cristina R.	Brazil
* Chapman	Jeremy	Australia
Chen	Zhonghua Klaus	People's Republic of China
Codreanu	Igor	Moldova
Cole	Edward	Canada
Cozzi	Emanuele	Italy
* Danovitch	Gabriel	USA
Davids	Razeen	South Africa
De Broe	Marc	Belgium
* De Castro	Leonardo	Philippines
* Delmonico	Francis L.	USA
Derani	Rania	Syria
Dittmer	Ian	New Zealand
Domínguez-Gil	Beatriz	Spain
Duro-Garcia	Valter	Brazil
Ehtuish	Ehtuish	Libya
El-Shoubaki	Hatem	Qatar
Epstein	Miran	United Kingdom
* Fazel	Iraj	Iran
Fernandez Zincke	Eduardo	Belgium
Garcia-Gallont	Rudolf	Guatemala
Ghods	Ahad J.	Iran
Gill	John	Canada
Glotz	Denis	France
Gopalakrishnan	Ganesh	India
Gracida	Carmen	Mexico
Grinyo	Josep	Spain
Ha	Jongwon	South Korea
* Haberal	Mehmet A.	Turkey
Hakim	Nadey	United Kingdom
Harmon	William	USA
Hasegawa	Tomonori	Japan
Hassan	Ahmed Adel	Egypt
Hickey	David	Ireland
Hiesse	Christian	France
Hongji	Yang	People's Republic of China
Humar	Ines	Croatia
Hurtado	Abdias	Peru
Ismail Moustafa	Wesam	Egypt
Ivanovski	Ninoslav	Macedonia
* Jha	Vivekanand	India
Kahn	Delawir	South Africa
Kamel	Refaat	Egypt
Kirpalani	Ashok	India
Kirste	Guenter	Germany
* Kobayashi	Eiji	Japan
Koller	Jan	Slovakia
Kranenburg	Leonieke	The Netherlands
* Lameire	Norbert	Belgium
Laouabdia-Sellami	Karim	France
Lei	Ruipeng	People's Republic of China
* Levin	Adeera	Canada
Lloveras	Josep	Spain
Lõhmus	Aleksander	Estonia
Luciolli	Esmeralda	France
Lundin	Susanne	Sweden
Lye	Wai Choong	Singapore
Lynch	Stephen	Australia
* Maïga	Mahamane	Mali
Mamzer Bruneel	Marie-France	France
Maric	Nicole	Austria
* Martin	Dominique	Australia
* Masri	Marwan	Lebanon
Matamoros	Maria A.	Costa Rica
Matas	Arthur	USA
McNeil	Adrian	United Kingdom
Meiser	Bruno	Germany
Meši	Enisa	Bosnia
Moazam	Farhat	Pakistan
Mohsin	Nabil	Oman
Mor	Eytan	Israel
Morales	Jorge	Chile
Munn	Stephen	New Zealand
Murphy	Mark	Ireland
* Naicker	Saraladevi	South Africa
Naqvi	S.A. Anwar	Pakistan
* Noël	Luc	WHO
Obrador	Gregorio	Mexico
Oliveros	Yolanda	Philippines
Ona	Enrique	Philippines
Oosterlee	Arie	The Netherlands
Oyen	Ole	Norway
Padilla	Benita	Philippines
Pratschke	Johann	Germany
Rahamimov	Ruth	Israel
Rahmel	Axel	The Netherlands
Reznik	Oleg	Russia
* Rizvi	S. Adibul Hasan	Pakistan
Roberts	Lesley Ann	Trinidad and Tobago
* Rodriguez-Iturbe	Bernardo	Venezuela
Rowinski	Wojciech	Poland
Saeed	Bassam	Syria
Sarkissian	Ashot	Armenia
* Sayegh	Mohamed H.	USA
Scheper-Hughes	Nancy	USA
Sever	Mehmet Sukru	Turkey
* Shaheen	Faissal A.	Saudi Arabia
Sharma	Dhananjaya	India
Shinozaki	Naoshi	Japan
Simforoosh	Nasser	Iran
Singh	Harjit	Malaysia
Sok Hean	Thong	Cambodia
Somerville	Margaret	Canada
Stadtler	Maria	USA
* Stephan	Antoine	Lebanon
Suárez	Juliette	Cuba
Suaudeau	Msgr. Jacques	Italy
Sumethkul	Vasant	Thailand
Takahara	Shiro	Japan
Thiel	Gilbert T.	Switzerland
* Tibell	Annika	Sweden
Tomadze	Gia	Georgia
* Tong	Matthew Kwok-Lung	Hong Kong
Tsai	Daniel Fu-Chang	Taiwan
Uriarte	Remedios	Philippines
Vanrenterghem	Yves F.C.	Belgium
* Vathsala	A.	Singapore
Weimar	Willem	The Netherlands
Wikler	Daniel	USA
Young	Kimberly	Canada
Yuldashev	Ulugbek	Uzbekistan
Zhao	Minggang	People's Republic of China

* = Members of the Steering Committee. (William Couser, USA, was also a member of the Steering Committee but was unable to attend the Summit.)
